# On the Use of Tobacco in Producing Sciatica

**Published:** 1853-05

**Authors:** Harvey Jewett

**Affiliations:** Canandaigua, N. Y.


BUFFALO MEDICAL JOURNAL

AND

MONTHLY REVIEW.

VOL. 8. MAY, 1853. NO. 12.

### ORIGINAL COMMUNICATIONS.

### ART. I. — *On the use of Tobacco in producing Sciatica.* By Harvey Jewett, M. D., Canandaigua, N. Y.

"The cold *Sciatica* cripples
Our senators, that their limbs may halt as lamely as their manners."

The positive and latent influence of tobacco in the production of disease, has never received its merited share of attention from investigating medical writers.

Scarcely a page in our standard authors, and only occasionally a floating paragraph in medical Journals, has called public attention to this most fruitful, and all-pervading source of human misery.

The universality of the practice of chewing and smoking tobacco, and our familiarity with its use, may in a measure explain the fact, that we have hitherto overlooked its undeniable influence in producing that class of affections denominated neuralgic.

The effects of tobacco upon the uninitiated, are so unmistakable, so real, and positive, that we need not stop to explain them.

If any doubt exists on this point, let the novitiate smoke, chew, or apply tobacco to the surface of the body for a short time, and the pallid face, the disgorged stomach, and palsied energies, will bear ample testimony to the truthfulness of our position.

So prompt and powerful are the effects upon the nervous system that it is not only unreasonable, but it is little short of madness to suppose that the habitual use of the narcotic for years, will cause it to be tolerated with impunity.

And strange as it may seem in view of the universally acknowledged effects of this poison, many medical men have not only recommended it to others for fancied or real diseases, but contrary to their own animal instincts, have vitiated their natural appetites, instilled into their persons a deleterious narcotic influence, and fallen into one of the most filthy habits that ever perverted the taste of a refined and cultivated people.

It is not our purpose, at the present time, to speak of the general, moral, and physical depravity, growing out of the use of tobacco, or even to allude to the numberless diseases which it engenders, but to confine our investigation to its effects upon the lower part of the spinal column.

That tobacco is instrumental, with certain constitutions and temperaments, in producing a large share of the limping, halting, cane-depending, crutch-using necessities so common among us, can be attested by a little observation on the part of the physician, and the result of his inquiries will essentially aid in relieving or radically curing those who suffer under these painful difficulties.

Diseases of the nervous system are universally acknowledged to be the most recondite and obscure of any class of affections to which the human system is liable.

The remote and proximate causes, and the unexplained mystery that hangs over the intense neuralgic suffering without any visible or appreciable change in the structure of the nerve itself, or any of the adjoining tissue, have been the theme of much fruitless speculation and inquiry.

The most intense agony is endured for years without the slightest morbid appearances; neither inflammation, thickening, adhesion of structure, nor the slightest deviation of any kind from the healthy condition of the part being apparent.

There is no part of the body which may not be the seat of neuralgic pains, yet some parts are more liable to them than others.

They are less frequently met with in the viscera supplied by the great sympathetic nerve than elsewhere.

Nervous pains are more common and severe in those parts which receive their nerves from the fifth pair, as the face and adjoining region.

In ascribing a large share of one class of neuralgic diseases to the influence of tobacco, it is not to be supposed that this is a tithe of the suffering and misery that tobacco entails upon its victims. A large proportion of those who use it keep themselves constantly under its narcotic influence. It pervades the whole animal economy, from the cerebral mass and spinal column, to the minutest nervous filament. All the organic functions are changed or modified by it, every drop of blood, every muscular fiber, every vitalizing breath, is to some extent brought under its modifying and pernicious influence.

In short, the head, the heart and the lungs, are made to feel directly the potential agency, and enduring consequences of this poison.

The brain is befogged, the blood is poisoned, the nervous system enervated, and the elements of disease scattered unconsciously throughout the delicate mechanism of the human frame.

There is great difference in the constitution and temperament of men, consequently the effects of tobacco will be varied, and more speedily manifested in some than in others.

Sciatica or lumbago, is not *necessarily the result* of excessive tobacco using. Neither does trembling of the hands, indigestion of food, palpitation of the heart, or indecision of purpose, follow in all cases as a consequence of this indulgence; but some, and perhaps many of the physical ills charged over to tobacco, will assuredly develop themselves in some form.

If in prosecuting our investigation, we find a large share of those addicted to the habit of smoking, subject to palpitation of the heart, disease of the throat, or sciatica, we are most naturally led to conclude that some connection, dependence or relation, exists between the habit and its almost unvarying consequences. And because we are unable to understand, or explain the phenomenon, it may be none the less true. If the attention of medical men should be turned to this subject, we believe the position might be established that nine-tenths of that form of neuralgic disease denominated sciatica or pain in the hip and leg, are the direct and legitimate effect of smoking or chewing tobacco.

It is true that only a small proportion of those who smoke are afflicted in this way, but our experience goes to prove, that disease of the nerves of the lower part of the spine and hip seldom exist except in connection with tobacco-using in some form, and generally in that of smoking. "A leading practitioner in England, gives a list of sixteen cases of *paralysis* produced by smoking, which came under his own knowledge within the last six months."

This is a significant fact, and goes to show the varied influence tobacco exerts upon different temperaments and constitutions.

Without going into a discussion of the subtle intricacies of nervous affections, and the manner in which external influences and vicious indulgences aid in the development of Sciatica or other neuralgic pains, I submit some statistics and facts taken from my note book, showing the connection between habitual tobacco-smoking and Sciatica and other anomalous ailments of the loins, hips, and lower extremities.

CASE I.—Mr. N——, aged 48, a farmer, of good constitution, and apparently robust health, of sallow, dingy complexion so common to smokers, applied for relief in the summer of 1852, stating that he suffered the most intense agony in his right hip and leg.

On investigating his case, I learned he had been complaining of rheumatism, as he expressed it, in his back, at different times, for several years; but had attended to his usual business up to within a few weeks of my seeing him.

His health was good aside from the pain in the hip, which was severe and uninterrupted, except when lying upon his back, when he was comparatively easy.

The usual remedies had been resorted to with no effect. On instituting a critical inquiry into his habits, I ascertained he had smoked from five to fifteen pipes-full daily for eighteen or twenty years.

Upon indicating to him the probable cause of his suffering, he promptly decided to make any sacrifice that would afford him relief. The pipe was laid aside, he gradually improved, and in two months was entirely free from pain. He called upon me a few days since, and expressed his gratification that his attention had been directed to the subject of smoking, that he was unwilling to believe himself so enslaved to the practice until he attempted to abstain from its use, and that he felt a degree of vivacity of spirit, and elasticity of body he had not known for many years.

CASE II.—Mr. G——, aged 68, a farmer, of bilious temperament, and swarthy, dusky complexion; had been accustomed to labor in the earlier part of his life, but was prematurely infirm and disabled on account of pain in his hip, which he had suffered, with more or less intensity, for twenty years.

He also had vertigo, palpitation, indigestion, sore mouth and tongue, together with great physical prostration and debility. The latter symptoms had been gradually increasing upon him for several years.

At the time I saw him in consultation with his attending physician, he was confined to his bed, dejected in spirit, and appeared like a man eighty years old. His pulse was faltering and intermittent, faculties lethargic, sleep disturbed and dreamy, epithelial investment of the mouth and tongue abraded. In short, the energies of the system and vital powers, were evidently prematurely waning, without apparent or obvious cause, with no organic disease, and no fever. The only satisfactory explanation of his case was to be found in the fact, that the patient had been for forty years one of the most inveterate tobacco-smokers in that region. His habit was to smoke from morning until night, except when taking his meals, and during divine service on Sunday, when he had a brief interval, but he seldom failed to light his pipe before leaving the sanctuary to return home.

The habit with him was so confirmed, his system so constantly narcotized, that he was restless and wretched, except when in the enjoyment of his pipe.

Upon stating to him my opinion as to the cause of his disease and disability, he frankly acknowledged his conviction that tobacco-smoking had insidiously and gradually brought him to his present condition. That for years, when he had allowed himself to reflect upon the subject, he had known that it was destroying him, yet he had never been able to resist the almost insatiable demand of his perverted appetite. With this acknowledgment upon his lips, and death in full view before him, he would call for his pipe and take a whiff or two at a time, which practice he continued up to the period of his death.

In this case, which is an extreme one, we have not only the pain of the back and hip so common to smokers, but we have a clear and full development of its pernicious influence throughout the entire system. The hips, tongue, mouth and fauces, were as red as scarlet, which, together with the palpitation and vertigo, he had suffered to some extent, many years past.

With this train of physical ills constantly pressing upon him; with an appreciating sense, and in view of the ultimate consequences, he persisted in the habit of smoking to the last, and died a victim to his indulgence.

Case III. — Mr. B——, aged 36 years, a farmer, of sanguino-bilious tem perament, was seized suddenly, during the winter of 1851, with an excruciating pain in the hip, extending down the leg, unattended with redness or swelling, which disabled him from attending to his ordinary duties. The only relief he could obtain, was by lying upon his back. His general health was good, save the local pain, which continued during the winter and most of the succeeding summer. The ordinary remedies and nostrums were resorted to, with but partial and temporary benefit.

On inquiry Mr. B. stated that he had been in the habit of chewing twelve pounds of tobacco annually, for several years, and smoked a pipe occasionally when it was convenient. He was never without his quid, except when asleep, and then he sometimes neglected or forgot to lay it aside.

Upon being apprised of the possible relation between his tobacco-using and his suffering, he decided to abandon his pipe altogether, and chew much less.

The pain gradually abated as his system was partially relieved from the influence of tobacco, and he is at present comparatively free from it, although he continues to chew moderately.

Case IV. — Mr. W——, aged 67, a laborer, had smoked a pipe thirty-five years. He had suffered intense pain in the right hip and leg many years, which disabled him for active labor. About three years since his attention was directed to a small tumor upon the lower lip, which gradually increased in size, attended with a stinging, creeping sensation. When I saw him in consultation with his attending physician, the sublingual and maxillary glands were enlarged, indurated and painful, with a well-developed epithelial cancerous ulceration of the under lip, of which he died in a few months.

This man not only smoked habitually, but he made use of the same pipe for months, without burning out or purifying with fire. The lips, salivary glands, and lining membrane of the mouth, being constantly subjected to the acrid influence of an old pipe, are not unfrequently the seat of cancerous disease.

Case V. — Mr. R——, aged 47, a farmer, of active habits, good constitution, and unusually robust appearance, was seized with severe pain in the loins and hip while attending court as a juror in May, 1851. The pain continued with great severity during the spring and summer, despite the nostrums and pathys that were resorted to for a cure. At times the pain was periodical, returning regularly every twenty-four hours with greater intensity and attended with spasmodic action of the muscles, so as to draw the leg suddenly up to the body. Large and repeated doses of morphine gave only partial relief from the suffering.

Much obscurity rested over the pathological character of the case. He was treated alternately during his illness for lumbago, psoas-abscess, and sciatica. Early in October there was observed on the left side of the spine, about the fourth lumbar vertebra, a slight prominence which gave evidence on pressure of something soft, doughy, or yielding, beneath the fascia — of indistinct, or obscure fluctuation. When standing upon his feet he inclined to lean backward to relieve himself of pain and tension produced by standing upright or bending forward. Upon consultation it was deemed advisable to puncture at the point of the elevation. A double-edged bistoury was introduced one inch and a half, when the resistance to the progress of the knife ceased as though it entered a cavity; but no discharge save florid blood could be obtained. The patient continued to suffer the most excruciating pain for a few weeks and died.

In making a post-mortem examination we found the center of the longissimus dorsi muscle filled with a whitish, cheesy, brain-like substance, which extended along the course of the spinal column and sacrum, to the gluteal muscles where it was found in globular masses from the size of a pea to that of an acorn. The center of the longissimus dorsi contained nearly a pint of the encapholoid deposit, while the exterior and adjacent structure was unchanged in appearance.

The transverse processes of the lower lumbar-vertebra were extensively diseased, involving the bodies of the bone.

A specimen of the diseased mass was submitted to microscopic test, by Prof. Dalton, which presented the characteristic evidence of tubercular formation.

Familiarity with the patient's habits for fifteen years, enables me to speak of them from personal observation. He was in the practice of smoking a pipe, most of his waking hours during that period of time. It was his constant companion when at his ordinary labor up to the time of the attack.

The question very naturally arises in the mind of the reader, had the habit of smoking any thing to do with the suffering and disease which caused his death. I have no hesitation in deciding affirmatively — and that he would not have been afflicted in that way had he never used tobacco.

The abruptness of the attack, is only one, among many instances that have fallen under my observation, when the subject had been in the habitual use of tobacco for a long period of time, with scarcely any perceptible manifestation of its untoward influence, until its insidious, silent, pent-up virulence, suddenly bursts upon its victim, revealing the occult treachery and deceitfulness of this pernicious practice.

Case VI. — Mrs. S——, aged 70, of bilious temperament and fine constitution; has had pain in the hip upward of twenty years, and is now unable to walk except by the aid of a crutch. She has smoked a pipe forty-five years.

CASE VII.—Mr. R——, aged 38, a farmer, athletic frame and unimpaired health, except the annoyance of pain in the hip and leg along the course of the ischiatic nerve. Mr. R. smoked a pipe most of the time when at his usual avocation, and all his leisure hours are occupied in that way.

CASE VIII.—Mr. A——, aged 72, has smoked a pipe 40 years; has had pain and lameness of the hip most of the time for thirty years past.

CASE IX.—Mr. H——, aged 35, has chewed tobacco fifteen years; has had pain in the hip and thigh, accompanied with lameness ten years.

CASE X.—Mr. B——, aged 49, a farmer; has smoked tobacco immoderately, twenty-six years; has had pain in one or both hips in the direction of the ischiatic nerve, fifteen years.

CASE XI.—Mrs. S——, aged 38, has been afflicted three years with pain along the track of the ischiatic nerve. Mrs. S. *never used tobacco.*

CASE XII.—Mr. L——, aged 60, a farmer, of large frame and apparent robust health; has smoked a pipe and tobacco thirty years; has had pain in his back and hip twenty years. And within the last five years has had two attacks of paralysis, from which he has partially recovered, although he continues to smoke occasionally.

CASE XIII.—Mr. J——, aged 45, has been in the habit of smoking a pipe twenty years, and has had pain in the hip eight years.

CASE XIV.—Mr. S——, aged 63, has smoked a pipe thirty-five years; pain in his back and hip twenty-two years.

CASE XV.—Mr. C——, aged 72, a farmer, had smoked a pipe forty-five years; has had pain in the hip twelve years, and is unable to walk without a cane.

CASE XVI.—Mr. J——, aged 28, had smoked alternately a pipe and segar nine years; had a severe attack of sciatica, followed by inflammation of the articular structure of the joint, with displacement of the head of the os femoris from the acetabulum.

Case XVII. — Mr. D——, aged 60 years, has smoked a pipe thirty-five years; had pain in hip and leg ten years.

Case XVIII. — Miss S——, a servant girl, aged 27, was suddenly seized with violent pain in the hip, in the latter part of the summer of 1852, after a hard day's work, and exposure to the evening air. She was sent to the poor-house of the county for treatment, where she still remains. She suffers severe pain in the direction of the ischiatic nerve, and is unable to walk without assistance. She has recently complained of pain in the chest, accompanied with cough and other symptoms of phthisis. The physical signs give undoubted evidence of tubercular formation in the summit of both lungs. *This patient never used tobacco.*

Case XIX. — Mr. P——, a farmer, aged 40, has had pain in the hip, running down the leg, four years; has smoked a pipe moderately twenty years.

Case XX. — Mr. N——, a laborer, aged 42, has smoked moderately, and chewed constantly, for twenty years. Has had pain in the right hip and leg three years.

Case XXI. — Mr. P——, aged 50, a painter by trade; has smoked twenty-five years; had pain in the hip four years. Still pursues his avocation and continues to smoke.

Case XXII. — Mr. A——, aged 48, of active business habits, and nervous temperament; has chewed tobacco twenty-five years. At one period he used three small papers of fine cut in a day; but for the last year or two he has chewed only two or three papers a week. He has been subject to attacks of what he calls neuralgic rheumatism; sometimes attacking the shoulders, arms and back, but the pain is most intense and continued in the direction of the ischiatic nerve. This case differs from most of the foregoing, inasmuch as the paroxysms of pain last but three or four weeks, and entirely disappear under treatment, and again recur at longer or shorter intervals.

The above twenty-two cases of this particular affection of the nerve of the hip, constitute all that have fallen under the writer's observation in a practice of twenty years.

Some of the subjects were advanced in life, and it may be said, were only suffering the rheumatic pains, and decrepitude incident to age. But in almost every instance, the suffering and lameness commenced in the prime of life, and before they had reached threescore, and ten were not only suffering, but disabled and dependent; unlike their cotemporaries in life, who had performed more labor without the use of tobacco, and were comparatively erect in person, and still active in habit. Two of the above cases, who still suffer pain along the course of the ischiatic nerve, never used tobacco in any form.

Many of the cases referred to are still living in the delightful enjoyment of the luxury of tobacco, notwithstanding the writhing agony and decrepitude they endure as the penalty of the indulgence.

Some of them, doubtless, ignorantly shortened their lives and greatly increased their suffering, by persistence in the practice of this irrational and fancied pleasure.

Three of the more recent cases under medical advice, were induced to abstain from the use of tobacco, and were relieved after a few months from the suffering in the hip, and restored to the enjoyment of health, which was unknown to them while under the influence of the narcotic.

That there is an incalculable amount of human wretchedness growing out of the enormous consumption of tobacco, no one of common observation will pretend to deny.

The profligate waste of health, time and money, the abridgment of precious lives, and many other considerations not within the legitimate scope of this paper, all combine to make it a subject worthy the most grave consideration by medical men.

CANANDAIGUA, April, 1853.

